# A phase II study on the role of gemcitabine plus romidepsin (GEMRO regimen) in the treatment of relapsed/refractory peripheral T-cell lymphoma patients

**DOI:** 10.1186/s13045-016-0266-1

**Published:** 2016-04-12

**Authors:** Cinzia Pellegrini, Anna Dodero, Annalisa Chiappella, Federico Monaco, Debora Degl’Innocenti, Flavia Salvi, Umberto Vitolo, Lisa Argnani, Paolo Corradini, Pier Luigi Zinzani, On behalf of the Italian Lymphoma Foundation (Fondazione Italiana Linfomi Onlus, FIL)

**Affiliations:** Institute of Hematology “L. e A. Seràgnoli”, University of Bologna, Via Massarenti, 9-40138 Bologna, Italy; Fondazione IRCCS Istituto Nazionale dei Tumori, Milan, Italy; Azienda Ospedaliera-Universitaria Città della Salute e della Scienza di Torino, Turin, Italy; A.O. SS Antonio e Biagio e Cesare Arrigo, Alessandria, Italy

**Keywords:** Peripheral T-cell lymphoma, Relapsed, Refractory, Gemcitabine, Romidepsin

## Abstract

**Background:**

There is no consensus regarding optimal treatment for peripheral T-cell lymphomas (PTCL), especially in relapsed or refractory cases, which have very poor prognosis and a dismal outcome, with 5-year overall survival of 30 %.

**Methods:**

A multicenter prospective phase II trial was conducted to investigate the role of the combination of gemcitabine plus romidepsin (GEMRO regimen) in relapsed/refractory PTCL, looking for a potential synergistic effect of the two drugs. GEMRO regimen contemplates an induction with romidepsin plus gemcitabine for six 28-day cycles followed by maintenance with romidepsin for patients in at least partial remission. The primary endpoint was the overall response rate (ORR); secondary endpoints were survival, duration of response, and safety of the regimen.

**Results:**

The ORR was 30 % (6/20) with 15 % (3) complete response (CR) rate. Two-year overall survival was 50 % and progression-free survival 11.2 %. Grade ≥3 adverse events were represented by thrombocytopenia (60 %), neutropenia (50 %), and anemia (20 %). Two patients are still in CR with median response duration of 18 months. The majority of non-hematological toxicities were mild and transient. No treatment-related death occurred and no toxicity led to treatment interruption.

**Conclusions:**

GEMRO combination regimen shows efficacy data similar to those of single-agent romidepsin with additional hematologic toxicities. Synergy observed in preclinical phase did not turn into ability to improve clinical outcomes.

**Trial registration:**

The trial was registered under EudraCT 2012-001404-38; ClinicalTrials.gov number, NCT01822886.

## Background

Peripheral T-cell lymphomas (PTCL) comprise a heterogeneous group of lymphoid malignancies arising from mature T-cells which present with different phenotypes and clinical presentations [1, 2]. These aggressive lymphomas are uncommon, constituting 10–12 % of non-Hodgkin’s lymphomas (NHL) in Western countries [3] but are relatively common in Eastern Asian, constituting about 20 % of mature NHL [4]. There is no consensus regarding optimal treatment for PTCL, especially in relapsed or refractory cases, which have very poor prognosis. High-dose chemotherapy followed by autologous stem cell transplantation (ASCT) has been accepted as a salvage treatment for eligible patients, although the evidence is unclear [5]. Moreover, the overall prognosis remains dismal in patients unsuitable for ASCT. Thus, effective salvage chemotherapy prior to ASCT or optimal therapeutic approach for patients ineligible for ASCT should be investigated in order to improve the prognosis of PTCL patients. Despite the enormous advances in our understanding of aggressive lymphomas, it is clear that progress in PTCL management has lagged well behind other B-cell malignancies. Over the past 5 years, the US Food and Drug Administration (FDA) has approved four drugs for patients with relapsed/refractory PTCL, and, counting the recent Japanese approval of the anti-CCR4 monoclonal antibody for patients with adult T-cell leukemia/lymphoma, five drugs have been approved worldwide [6–10]. These approvals have led to the initiation of no fewer than four randomized clinical studies, exploring the integration of these new agents into standard CHOP (cyclophosphamide–adriamycin–vincristine–prednisone)-based chemotherapy regimens for patients with newly diagnosed PTCL [11–14]. In addition, new waves of studies are exploring the potential benefits of novel drug combinations, an effort to build on the obvious single-agent successes. What has emerged most recently is the recognition that PTCL may be a disease characterized by epigenetic dysregulation: this could explain PTCL sensitivity to histone deacetylase (HDAC) inhibitors and open the door for even more creative combination approaches.

Gemcitabine (2′,2′-difluorodeoxycytidine) is a pyrimidine anti-metabolite with clinical activity in aggressive lymphomas. The overall response rates (ORR) for gemcitabine as a single agent in relapsed/refractory PTCL are up to 50 % [15, 16], while the single-agent activity is lower in relapsed/refractory aggressive B-cell NHL with a reported ORR of 20 % in small series [17]. Gemcitabine-based combinations with other chemotherapeutic agents afford higher response rates, although even toxicity occurrence is higher (especially myelosuppression and infective complications) [18].

Romidepsin is an HDAC inhibitor that was approved by FDA in 2011 for the treatment of PTCL in patients who have received at least one prior therapy [7]. A larger, pivotal phase II study on romidepsin in patients with relapsed or refractory PTCL showed an overall response rate of 25 % (complete response [CR] rate of 15 %) without significant differences in response rates between patient subgroups that included major PTCL subtypes [7]. On the basis of these data, there are some trials examining the combination of romidepsin with conventional chemotherapy regimens [11, 19]. Currently, romidepsin is under investigation for patients with PTCL in various combinations, i.e., aurora A kinase inhibitor alisertib [20], proteasome inhibitors bortezomib [21], carfilzomib [22], lenalidomide [23, 24], and pralatrexate [25].

Starting from these data and due to the still unmet clinical need for patients with relapsed/refractory PTCL, we designed a multicenter phase II study to investigate the role of the combination of gemcitabine plus romidepsin (GEMRO regimen) looking for a potential synergistic effect of the two drugs.

## Methods

### Study population and eligibility criteria

Patients with histologically proven PTCL as per WHO criteria and for whom previous treatments had failed were eligible for this study. Additional inclusion criteria included measurable disease, an absolute neutrophil count >1 × 10^9^/l, hemoglobin >8 g/dl, platelets >100 × 10^9^/l, and normal renal and hepatic functions. All patients had to have an Eastern Cooperative Oncology Group performance status (PS) score of 0–2 at time of enrolment. Patients had not to have received prior gemcitabine or romidepsin therapy.

### Study design

This was a phase II clinical study on patients with PTCL conducted at 4 Italian centers. The local ethic committee at each centre approved the study protocol and its amendments, in accordance with the Italian law and complying with the Declaration of Helsinki (ethical committee of the coordinating center, Comitato Etico Policlinico S. Orsola-Malpighi Bologna, Italy; reference number 045-2012/U). Patients provided written informed consent before enrolment.

The dose and schedule of this combined regimen were extrapolated by a phase I trial of romidepsin in combination with gemcitabine in patients with pancreatic and other advanced solid tumors [26]. The present phase II study included an induction part with romidepsin 12 mg/m^2^ i.v. on days 1, 8, and 15 and gemcitabine 800 mg/m^2^ i.v. on days 1 and 15 for 6 cycles, each cycle to be repeated every 28 days. After this induction phase, patients who obtained at least a partial response (PR) proceeded onto romidepsin maintenance at the dose of 14 mg/m^2^ i.v. until disease progression.

Clinical evaluations at the time of study entry included medical history and physical examination, complete blood cell count, serum biochemistry, electrocardiogram and echocardiogram, computed tomography (CT) scan of the neck, chest, abdomen, and pelvis, and positron emission tomography (PET) scan of the total body. Bone marrow and lymph node biopsies had not to be if performed before signing the study informed consent as part of standard medical care within 56 days before GEMRO for bone marrow biopsy and within 6 months for lymph node biopsy, respectively.

Patient response was evaluated after 3 cycles of GEMRO and 4 weeks after the end of the induction phase (i.e., after the sixth cycle); in addition, during the maintenance phase with romidepsin, the response evaluation was done every 4 months for the first 2 years. All sites of initial disease were reassessed by CT scan, PET scan, and bone marrow biopsy for patients who had bone marrow involvement. Response assessment was performed according to the Revised Response Criteria for Malignant Lymphoma [27].

The trial was registered under EudraCT 2012-001404-38; ClinicalTrials.gov number, NCT01822886.

### Statistical analysis

Sample size estimation was performed by Fleming’s single-stage procedure. Defined p0 as the proportion of response below which the treatment does not warrant further investigations and pα as the proportion of responses beyond which a phase 3 trial should be carried out, we set p0 = 0.3 and pα = 0.7. The number of patients required, given a type I error at 0.05 one sided and a power of 80 %, was 18 and the number of successes 13. Taking into account a dropout of 10 %, the number of patients was set at 20.

The primary endpoint of this study was ORR after the induction phase, consisting of the sum of CR and PR rates; the secondary endpoints were duration of the response, progression-free survival (PFS), overall survival (OS), and safety of the GEMRO regimen [27]. Survival curves were estimated using the Kaplan-Meier method. For the safety analyses, frequency of toxicities was reported by type and grade according to the National Cancer Institute Common Terminology Criteria for Adverse Events (version 4.0).

Efficacy and safety were evaluated on the basis of intention-to-treatment. All analyses were performed using STATA (version 11.1).

## Results

### Patient characteristics

A total of 20 patients with relapsed/refractory PTCL were enrolled and treated between January 2013 and December 2014. The baseline characteristics of the patients in this study are summarized in Table 1. The median age of patients was 55 years (range 24–77 years), and 10 were males (50 %). All patients had a good PS of 0 or 1. Regarding the histology, 10 (50 %) patients had PTCL not otherwise specified (PTCL-NOS), 9 (45 %) patients had angioimmunoblastic T-cell lymphoma (AITL), and 1 (5 %) patient had anaplastic large cell lymphoma (ALCL) anaplastic lymphoma kinase (ALK) negative. No follicular helper T-cell cases were observed. One patient (5 %) was stage II, and 19 patients (95 %) were stage III or IV. In addition, there were 10 patients (50 %) with extranodal involvement, 6 of whom with bone marrow involvement. The 80 % of subjects had an international prognostic index score ≥2. Patients had received a median of two (range, 1–4) prior treatment regimens and 7 (35 %) of patients had failed prior autologous stem cell transplantation. There were 12 refractory cases (60 %) and 8 relapsed cases (40 %).

**Table 1:** Patient characteristics

Characteristic	Patients
	*n* (%)
Age, years	
Median	55
Range	24–77
Sex	
Male	10 (50)
Female	10 (50)
Histology	
PTCL-NOS	10 (50)
AITL	9 (45)
ALCL, ALK negative	1 (5)
Stage at enrolment	
I–II	1 (5)
III–IV	19 (95)
Extranodal involvement	10 (50)
International Prognostic Index	
≥2	16 (80)
No. of prior regimens	
Median	2
Range	1–4
Refractory to most recent therapy	12 (60)

*PTCL-NOS* peripheral T-cell lymphoma not otherwise specified, *AITL* angioimmunoblastic T-cell lymphoma, *ALCL* anaplastic large cell lymphoma, *ALK* anaplastic lymphoma kinase

### Response to treatment and outcomes

Patients started GEMRO therapy at a median time from diagnosis of 13.9 months. The median number of cycles received per patient during induction phase was 3 (range 1–6). Six patients withdrew from treatment before the first scheduled restaging due to objective clinical progression of the underlying disease.

**Fig. 1:**
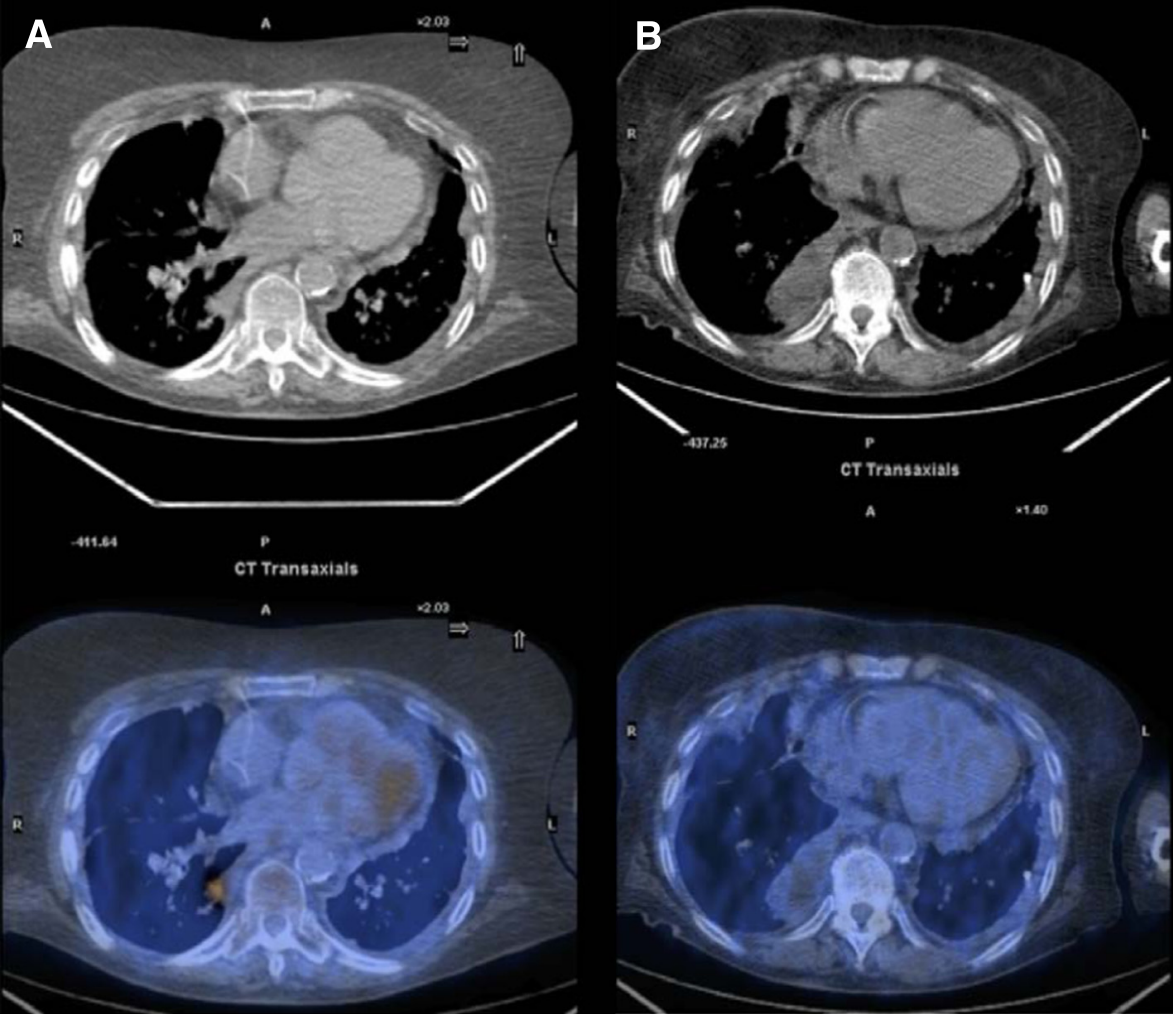
Clinical case: positron emission tomography results pre- (**a**) and post-therapy (**b**)

At the end the induction phase, the ORR was 30 % (6/20 patients) and included 3 (15 %) CR and 3 (15 %) PR; among the remaining patients, stable disease was observed in one (5 %) case and 13 (65 %) had progressive disease. According to the histologic subsets, among the 6 responders, there were 4 PTCL-NOS (2 CR and 2 PR) and 2 AILT (1 CR and 1 PR). One (an AITL) of the 3 CR patients discontinued the treatment after 4 cycles due to cardiologic problems not related to the therapeutic regimen, and she is currently in CR after 35 months: Fig. 1 shows the rapid and complete disappearance of the lymphoma assessed by PET scan.

All the responders (CRs and PRs), except for the CR patient who stopped the treatment after 4 cycles, underwent maintenance phase with romidepsin. Current median number of cycles in the maintenance phase is 5, but one patient is still on treatment (12th cycle).

During the maintenance period, two PRs had a disease progression after 4 and 5 months, respectively; the third PR patient, after 2 months of maintenance, underwent allotransplant obtaining CR. At this time, 2/3 CRs are in continuous response after 12 and 35 (AILT patient treated with only 4 cycles) months, respectively. One CR patient relapsed after the sixth cycle of maintenance. Median duration of response was 12.4 months, 18 months for CR patients.

**Fig. 2:**
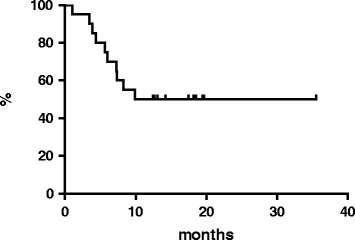
Overall survival

**Fig. 3:**
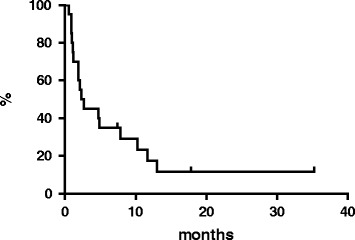
Progression-free survival

At a median follow-up of 18 months, the 2-year OS rate for all patients was 50 % (median reached at 22 months, Fig. 2), the 2-year PFS rate for all patients was 11.2 % with median reached at 2.5 months (Fig. 3). At the latest follow-up, 10 patients were deceased due to lymphoma and 10 patients were still, 3 of whom without disease.

### Safety

Treatment-related toxicities are shown in Table 2. The most common grade 3 or 4 toxicity was thrombocytopenia, which was observed in 12 patients (60 %); grade 3 or 4 neutropenia was observed in 10 (50 %) patients while grade 3/4 anemia occurred in 20 % of cases. The majority of non-hematological toxicities were mild and transient. There was no grade 4 non-hematological toxicity observed in the study; however, a grade 3 transaminase increase occurred in 3 patients. No treatment-related death occurred and no toxicity led to treatment interruption.

**Table 2:** Treatment-related adverse event: all events and grade ≥3 events

	Any grade	Grades 3–4
	*N* (%)	*N* (%)
Anemia	12 (60)	4 (20)
Neutropenia	11 (55)	10 (50)
Thrombocytopenia	16 (80)	12 (60)
Febrile neutropenia	2 (10)	0
Nausea and vomiting	10 (50)	0
Pyrexia	6 (30)	0
Transaminase increase	4 (20)	3 (15)

## Discussion

The generally poor outcomes observed in PTCL patients highlight the urgent need for alternative treatment strategies. Several novel approaches have been evaluated in single-arm phase I and II studies, mainly in patients with relapsed/refractory disease, which has a particularly poor prognosis. In a recent registry study [28], PTCL patients receiving CHOP or CHOP-like regimens as first-line therapy showed poor outcomes: 25 % of patients had refractory disease with a median OS of 2.5 months and 53 % of responding patients relapsed with a median OS of 6 months. There is, consequently, a requirement for effective second-line treatments for relapsed and refractory PTCL. Over recent years, several single-agent therapies have proven to be effective in this setting. Pralatrexate, romidepsin, and belinostat are all approved broadly for PTCL with ORRs in large phase II studies of 29, 25, and 26 %, respectively [6, 7, 9]. Brentuximab vedotin is also approved in relapsed ALCL with an ORR of 86 % in a small phase II trial [8]. In addition, mogamulizumab (approved only in Japan) reported an ORR of 34 % in a small PTCL phase II study [10].

Regarding the combination of romidepsin and gemcitabine, there were some interesting preclinical data in solid tumors: romidepsin synergistically inhibits cell proliferation with gemcitabine by suppressing removal of incorporated harmful nucleotide analogues of DNA [29]. Despite minimal clinical activity of this combination in solid tumors [26], romidepsin plus gemcitabine is being studied in several phase I trials underway in patients with PTCL [30, 31].

In our study, we reported an ORR of 30 % with a CR rate of 15 %. The median duration of response was 12 months (range, 9–35 months with median follow-up of 18 months) and the median PFS was reached at 2.5 months. In responding patients, the achievement of CR was associated with prolonged PFS and OS compared with all other outcomes. The safety profile was overall hematologic, and particularly it was represented by thrombocytopenia and neutropenia.

The ORR and CR rates did not differ from data reported on romidepsin as a single agent [7]. At the same time, there was a similar outcome for responding patients as observed in the pivotal phase II study on romidepsin in monotherapy [32]. Remarkably, the median PFS was analogous to those reported for PTCL after relapse or progression, regardless of the treatment, e.g., romidepsin or belinostat [7, 9, 33]. On the other side, the toxicity profile of GEMRO regimen reported additional hematologic toxicities, as previously stated in the phase I study on solid tumors [26].

One possible reason for these disappointing results could be that cases with extensive and refractory disease were enrolled: 95 % of patients had stage III–IV disease and 60 % patients were refractory. It could be important to revise the GEMRO regimen on the basis of different schedule (dose and timing) of both drugs.

## Conclusions

We identified disappointing clinical results with GEMRO plan in relapsed/refractory PTCL. In fact, these preliminary data failed to show a superiority of the GEMRO combination regimen over single-agent romidepsin as salvage treatment in this setting of patients. These data could indicate that synergy observed in preclinical phase does not always turn into ability to improve clinical outcomes. For the next steps, potential modifications of the treatment schedule are requested to allow more substantial delivery of the treatment and subsequent better clinical response.

## Abbreviations

AITL: angioimmunoblastic T-cell lymphoma
ALCL: anaplastic large cell lymphoma
ALK: anaplastic lymphoma kinase
ASCT: autologous stem cell transplantation
CHOP: cyclophosphamide–adriamycin–vincristine–prednisone
CR: complete response
CT: computed tomography
FDA: Food and Drug Administration
HDAC: histone deacetylase
NHL: non-Hodgkin lymphoma
ORR: overall response rate
OS: overall survival
PET: positron emission tomography
PFS: progression-free survival
PR: partial response
PS: performance status
PTCL: peripheral T-cell lymphoma
PTCL-NOS: peripheral T-cell lymphoma not otherwise specified
